# Prediction of B cell epitopes in proteins using a novel sequence similarity-based method

**DOI:** 10.1038/s41598-022-18021-1

**Published:** 2022-08-12

**Authors:** Alvaro Ras-Carmona, Alexander A. Lehmann, Paul V. Lehmann, Pedro A. Reche

**Affiliations:** 1 Laboratory of Immunomedicine, Department of Immunology, Faculty of Medicine, University Complutense of Madrid, Pza Ramón y Cajal, s/n, 28040 Madrid, Spain; 2 Research and Development Department, Cellular Technology Limited (CTL), Shaker Heights, OH 44122 USA

**Keywords:** Computational models, Protein analysis, Applied immunology, Humoral immunity, B cells

## Abstract

Prediction of B cell epitopes that can replace the antigen for antibody production and detection is of great interest for research and the biotech industry. Here, we developed a novel BLAST-based method to predict linear B cell epitopes. To that end, we generated a BLAST-formatted database upon a dataset of 62,730 known linear B cell epitope sequences and considered as a B cell epitope any peptide sequence producing ungapped BLAST hits to this database with identity ≥ 80% and length ≥ 8. We examined B cell epitope predictions by this method in tenfold cross-validations in which we considered various types of non-B cell epitopes, including 62,730 peptide sequences with verified negative B cell assays. As a result, we obtained values of accuracy, specificity and sensitivity of 72.54 ± 0.27%, 81.59 ± 0.37% and 63.49 ± 0.43%, respectively. In an independent dataset incorporating 503 B cell epitopes, this method reached accuracy, specificity and sensitivity of 74.85%, 99.20% and 50.50%, respectively, outperforming state-of-the-art methods to predict linear B cell epitopes. We implemented this BLAST-based approach to predict B cell epitopes at http://imath.med.ucm.es/bepiblast.

## Introduction

A B cell epitope, also known as antigenic determinant, is defined as the specific portion of antigen that is recognized by the B cell receptor or its soluble form (antibodies) secreted after B cell activation^1–3^. B cell epitopes can be classified as conformational (also known as discontinuous) or linear (also known as continuous). In proteins, conformational B cell epitopes include residues that are not sequential in the primary structure, but close in space in the antigen three-dimensional structure^3,4^. In contrast, linear B cell epitopes consist of sequential amino acid residues. These B cell epitopes can be recognized by antibodies out of the remaining protein context and can replace the whole protein for antibody production^3,4^. There are numerous approaches and methods to predict linear B cell epitopes^3–7^. Some of them are based on amino acid propensity scales that depict physicochemical properties of B cell epitopes. The first of such scales was introduced by Hopp and Woods^8^ and many other scales followed latter, including those based on flexibility^9^, hydrophobicity^10,11^, surface accessibility^12^ and antigenicity^13^. Most recent approaches to predict B cell epitopes use machine learning algorithms such as neural network^14,15^, support vector machine^16–18^ or random forest^19,20^, which are trained on features of known B cell epitopes. As shown by various benchmark evaluations^21–23^, the performance of B cell epitope prediction methods can be quite low and there is still room for improvement.

B cell prediction methods rely on the existence of B cell epitope sequence commonalities. Thereby, we developed a sequence-similarity based method to predict linear B cell epitopes in protein sequences, using the basic local alignment search tool (BLAST)^24^. The approach relies in finding BLAST hits to a database including 62,730 known linear B cell epitopes extracted from the Immune Epitope DataBase (IEDB)^25,26^. For evaluation, any ungapped sequence hit to this database with identity ≥ 80% and length ≥ 8 was considered a B cell epitope. In tenfold cross-validations, this method reached an accuracy > 70% and > 65% in various independent datasets including B cell epitopes obtained from the BCIPEP database^27^ and different types of non-B cell epitopes, outperforming related methods such as those implemented by BepiPred^19,20^, IBCE-EL^28^ and LBtope^17^. BLAST-based B cell epitope predictions are available at BepiBlast (http://imath.med.ucm.es/bepiplast).

## Results

### B cell epitope BLAST database

**Figure 1:**
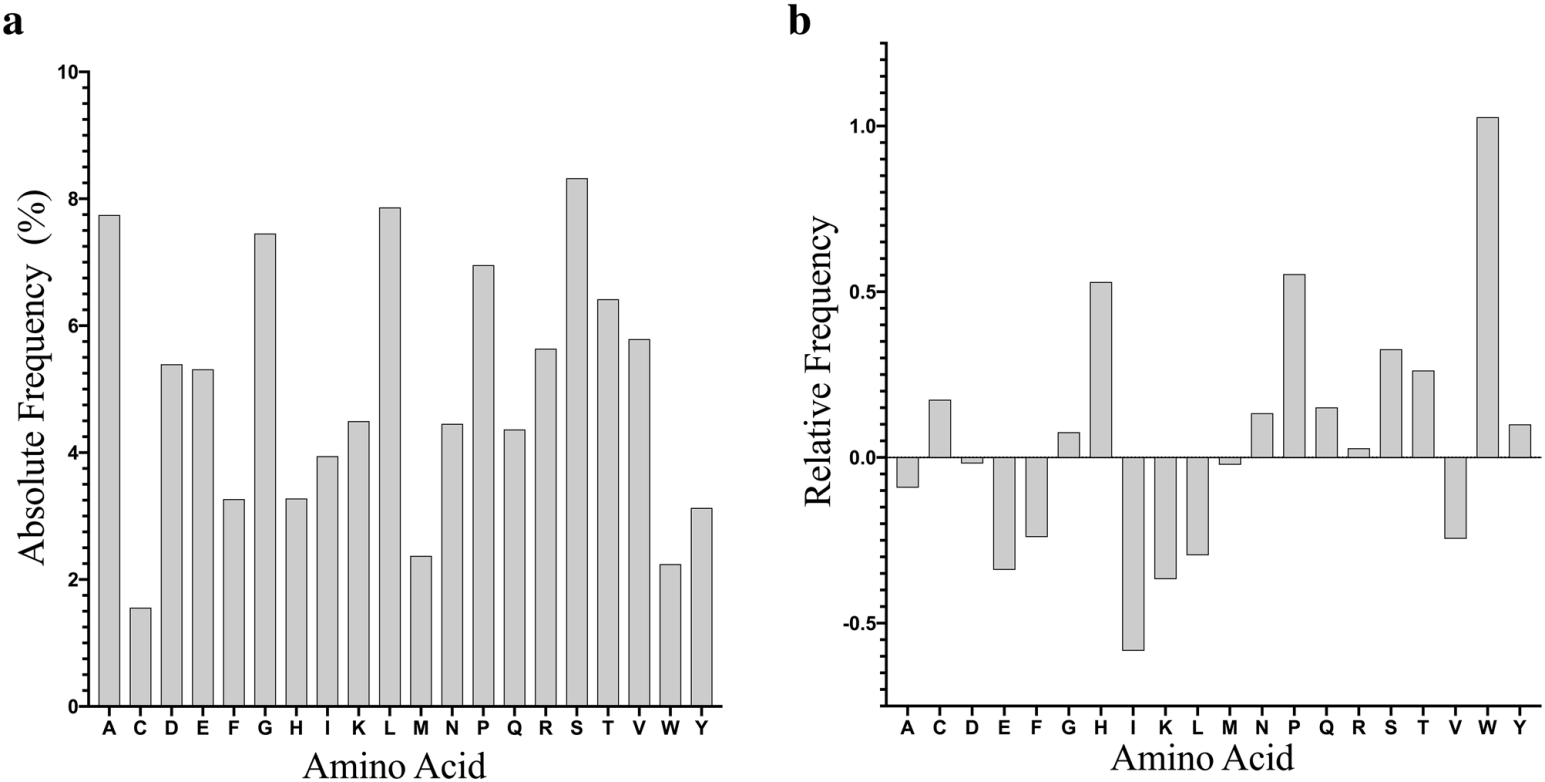
Absolute and relative amino acid frequencies in B-cell epitopes. (**a**) Figure shows the frequency in percentage (Y axis) of each of the 20 distinct amino acids (X axis) in B cell epitopes included in BEPIBD. (**b**) Figure represents the same amino acid frequencies but relative to those in SWISSPROT, represented as log2 values.

We built a BLAST formatted database upon the amino acid sequence of 62,730 experimentally verified linear B cell epitopes, including 940 that are known to be targeted by neutralizing antibodies, obtained from IEDB^25,26^. Hereafter we will refer to this database as BEPIBD. All B cell epitope sequences in BEPIBD range from 8 to 25 residues. BEPIBD is available as supplementary data at the journal website (Supplementary Dataset 1 online). The mean and median length of B cell epitope sequences in BEPIBD is 13.91 ± 2.85 and 12, respectively. Sequences included in BEPIBD did not share more than 90% identity and the average sequence similarity in the dataset was of 18.36 ± 6.17%. An amino acid frequency analysis revealed that some amino acids are more frequent than others in B cell epitopes (Fig. 1a). The most frequent amino acids are serine (8.33%), alanine (7.75%), leucine (7.87%) and glycine (7.45%), while cysteine (1.56%), tryptophan (2.24%) and methionine (2.37%) are less frequent. However, this scenario changed when amino acid frequencies in B cell epitopes were compared with those in SWISSPROT^29,30^. As shown in Fig. 1b, tryptophan, proline and histidine are in this case clearly overrepresented in B cell epitopes.

### BLAST discrimination of B cell epitope

We used BEPIBD as a target database for testing the ability of BLAST to discriminate between B and non-B cell epitopes as indicated in “Methods”. Briefly, we considered as a B cell epitope any query peptide with at least an ungapped hit with length ≥ 8 and identity ≥ 80% to BEPIBD. We evaluated this approach to discriminate B cell epitopes from non-B cell epitopes under tenfold cross-validation, considering various datasets of non B cell epitopes and the same dataset of B cell epitopes (details in “Methods”). Two negative datasets of non-B cell epitopes, RANDPEP and IEDBNB, each including 62,730 peptide sequences, were used in these tenfold cross-validation. RANDPEP includes peptides with random amino acid sequences and IEDBNB includes peptides with reported negative B cell epitope assays obtained from IEDB (more details in “Methods”). RANDPEP and IEDBNB are available as supplementary data at the journal’s website (Supplementary Datasets 2 and 3). The performance of the BLAST approach to discriminate B and non-B cell epitopes was determined by computing the sensitivity (SE), specificity (SP), accuracy (ACC) and the Matthew’s correlation coefficient (MMC) during tenfold cross-validations. As summarized in Table 1, the approach yielded an ACC of 72.54 ± 0.27% when considering as non-B cell epitopes those in the IEDBNB dataset and 81.32 ± 0.20% when considering the non-B cell epitopes in the RANDPEP dataset.

**Table 1:** Performance of BLAST-based discrimination of B and non-B cell epitopes.

Negative dataset	% SE	% SP	% ACC	MCC
RANDPEP	63.49 ± 0.43	99.15 ± 0.15	81.32 ± 0.20	0.67 ± 0.01
IEDBNB	63.49 ± 0.43	81.59 ± 0.37	72.54 ± 0.27	0.46 ± 0.01

Table reports the sensitivity (% SE), specificity (% SP), accuracy (% ACC), and Matthew’s correlation coefficient (MMC) of BLAST-based discrimination of B cell epitopes in BEPIBD from non-B cell epitopes included in the RANDPEP and IEDBNB datasets. Values were obtained in tenfold cross-validation experiments.

We also evaluated the BLAST-based method in an independent dataset of B and non-B cell epitopes using BEPIBD as the target for BLAST searches and compared the predictions with those produced by BepiPred^19,20^, IBCE-EL^28^, and LBtope^17^. B cell epitopes in the independent dataset consisted of 503 linear B cell epitopes obtained from the BCIPEP database^27^ (BECIP dataset). As before, we also considered two negative datasets, each including 503 non-B cell epitopes, consisting of random peptide sequences (IRPEP dataset) and non-B cell epitopes from IEDB (INB dataset), respectively, that were obtained as described previously but did not overlap with any of datasets previously described (BEPIBD, RANDPEP and IEDBNB). The global sequence similarity between BECIP dataset and the BEPIBD is of 17.86 ± 5.66% while the sequence similarity between the IRPEP and INB datasets and their counterparts, RANDPEP and IEDBNB, is of 18.21 ± 4.70% and 18.92 ± 6.10%, respectively. The BECIP, IRPEP and INB datasets are available as supplementary data at the journal’s website (Supplementary Dataset 4, 5 and 6).

The results of the B cell epitope predictions using the BLAST-based method, BepiPred, LBtope and IBCE-EL in the BECIP independent dataset in combination with two noted negative datasets are shown in Table 2. The measures of the performance achieved by the BLAST-based method in these independent tests were similar to those obtained in cross-validation (Table 1) but were a bit lower. Thus, the ACC achieved when considering random peptides as non-B cell epitopes (IRPEP peptides) was 74.85%, while in cross-validation was 81.32 ± 0.20%. Similarly, the ACC obtained considering non-B cell epitopes in the INB dataset was 69.48%, lower that the obtained in cross-validation (72.54 ± 0.27%). In any case, the BLAST-based method consistently outperformed all the competing methods in terms of ACC and MCC values in all the tests. The only noted exception was obtained on the INB dataset (non-B cell epitopes from IEDB) with the method IBCE-EL. In sum, this comparison underlines the ability of the BLAST-based method to predict B cell epitopes.

**Table 2:** Comparative performance of B cell epitope prediction methods.

Negative dataset	Method/tool	% SE	% SP	% ACC	MCC
IRPEP	BLAST	50.50	99.20	74.85	0.57
	BepiPred	37.60	65.01	51.35	0.03
	LBtope	42.21	76.34	59.32	0.20
	IBCE-EL	77.80	14.91	46.26	− 0.09
INB	BLAST	50.50	88.47	69.48	0.42
	BepiPred	37.60	66.60	52.14	0.04
	LBtope	42.41	77.73	60.02	0.20
	IBCE-EL	77.80	82.11	79.96	0.60

Table reports the sensitivity (% SE), specificity (% SP), accuracy (% ACC) and Matthew’s correlation coefficient (MMC) of the BLAST-based method, BepiPred, LBtope and IBCE-EL discriminating B cell epitopes in the BECIP dataset from non-B cell epitopes in two different datasets (IRPEP and INB). B cell epitope predictions with LBtope and IBCE-EL were carried out at the relevant web sites and BepiPred predictions were carried out using the standalone version of BepiPred (details in “Methods”).

### BepiBlast web server

**Figure 2:**
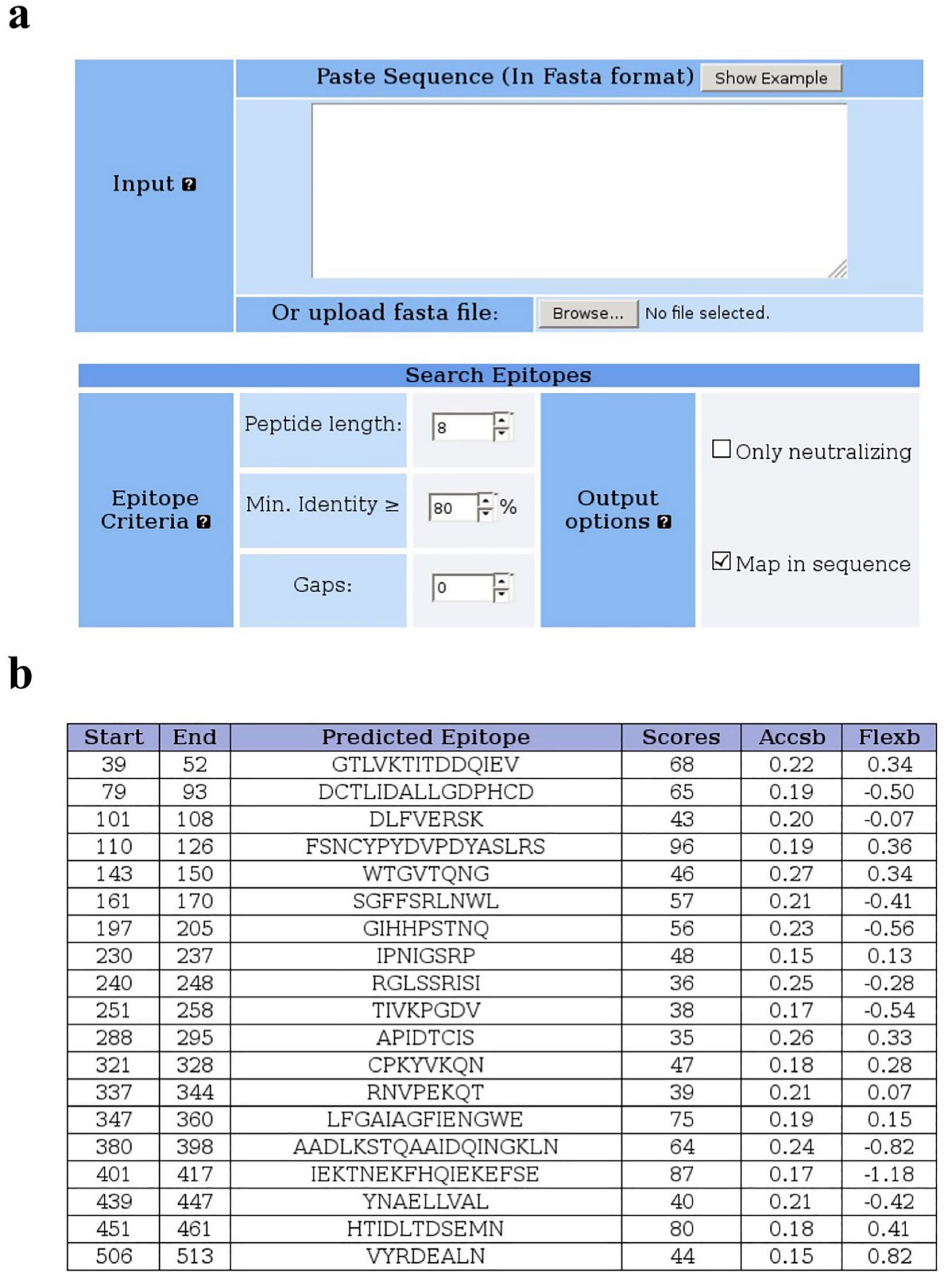
BepiBlast web server. (**a**) BepiBlast interface. (**b**) Representative BepiBlast output obtained with default settings. The shown results were obtained for hemagglutinin from Influenza A virus (UniProt Id: P03437). BepiBlast main result consists of a table displaying the following information (from left to right): peptide starting position; peptide ending position; predicted B cell epitope; bit score; accessibility value and flexibility value.

We have developed a web-based tool under the name of BepiBlast to enable the prediction of linear B cell epitopes using BLAST. BepiBlast is available for free public use at http://imath.med.ucm.es/bepiblast. The BepiBlast interface, shown in Fig. 2a, has been designed for intuitive and easy use. The input data for BepiBlast can be one or several protein sequences in FASTA format, which can be pasted or uploaded to the server. After submission, BepiBlast runs a BLASTP against BEPIBD and processes the BLAST output to identify B cell epitopes as query fragments from ungapped hits with identity and length higher than 80% and 8, respectively. These search criteria to identify B cell epitopes within protein queries (gaps, minimum identity and peptide length) can be modified by the user. Moreover, if the option “Only neutralizing” is selected, BepiBlast will only return B cell epitopes resulting from hits to neutralizing epitopes. The main output of BepiBlast (Fig. 2b) consists of a table listing all non-overlapping B cell epitopes with bit scores and predicted accessibility and flexibility computed as indicated in “Methods”. Since BLAST searches often detect overlapping B cell epitope hits, BepiBlast uses *Z*_*b*_ values, computed as indicated in “Methods”, to simplify the results and return non-overlapping B cell epitope cores. Likewise, BepiPred uses *Z*_*b*_ values to color the sequence residues into an RGB scale and visualize the predicted B cell epitopes in the query sequence. Such visualization is shown if the “Graphics” option is selected. The output of BepiBlast also includes BLAST hit information, including the source or the IEDB ID of the known epitope, which is processed to return the predicted B cell epitopes.

## Discussion

Determining the specific regions of a protein that can be recognized by antibodies, B cell epitopes, is of great practical interest. In fact, the primary aim of predicting B cell epitopes in protein sequences is to identify constituent fragments that can substitute the entire protein to produce specific antibodies. In this sense, predicting linear B cell epitopes is of particular relevance for they can be formulated as synthetic peptides which are suitable for antigen-specific antibody production. Currently, there are numerous methods and tools to predict linear B cell epitopes^3–7^. Given the essentially endless diversity of the BCR and antibodies, almost any peptide can be suitable for recognition and hence be a B cell epitope. Therefore, the most complex and recent B-cell prediction methods make use of machine learning (ML)-based models that are generated by training ML algorithms on feature data drawn from experimentally determined B cell epitopes and assumed non-B cell epitopes^14–20^. As a result of training, ML-algorithms capture subtle patterns into a single model that serve to distinguish B cell epitopes from non-B cell epitopes. Unfortunately, these approaches suffer from the fact that we do not have *bona fide* sets of non-B cell epitopes. Subsequently, ML-algorithms are generally trained on random peptides^14,19,31–33^ or peptides with reported negative B cell epitope assays^17,28,34^. However, it is questionable that random peptides, or peptides with reported negative assays, are not or cannot be antigenic. Not surprisingly, independent benchmark comparisons of B cell epitope prediction methods show that ML-based approaches are marginally better than simple amino acid propensity scales^21–23^. Given the noted limitations, in this work we explored an alternative B ell epitope prediction approach that only takes in consideration the large wealth of known B cell epitope sequences.

The IEDB, the largest repository of immune epitopes, currently includes more than 200,000 unique B cell epitope sequences (release 201,439). Thereby, we considered that a valid approach to predict B cell epitopes in protein sequences is to detect sequence similarities to individual known B cell epitopes using BLAST. To validate such approach, we gathered from IEDB a dataset of 62,730 known B cell epitopes to generate BLAST-formatted databases and considered a B cell epitope any BLAST hit with a length and identity ≥ 8 and 80%, respectively. We showed that in tenfold cross-validation this BLAST-based method could distinguish known B cell epitopes from two types of assumed non-B cell epitopes with a remarkable accuracy (Table 1). For example, the accuracy obtained considering non-B cell epitopes with reported negative assays is 72.54 ± 0.27%. We also showed that the accuracy of the BLAST-based method on an independent source of known B cell epitopes and two distinct datasets of non-B cell epitopes is above 69%, higher than that obtained with competing ML-based methods, such as BepiPred, IBCE-EL and LBtope (Table 2). There is however an exception. IBCE-EL achieved better accuracy than the BLAST method (79.96% vs 69.48%) when considering as non-B cell epitopes peptides with reported B cell epitope negative assays. However, it is worth noting that IBCE-EL models were precisely trained on such data. In other words, the testing dataset is a valid independent dataset to assess the accuracy of our method but not that of IBCE-EL.

Following the noted results, we developed a web-based tool, BepiBlast, enabling B cell epitope predictions in protein sequences using this BLAST-based method. Given the practical relevance, prediction of linear B cell epitopes has been tackled through numerous approaches, ranging from simple amino acid propensity scales to sophisticated models resulting of combining perturbation theory and machine learning^35–37^. Moreover, there are a number of tools to predict linear B cell epitopes that are available for free public use online (Table 3). In general, state-of-the-art tools for linear B cell epitope prediction implement alignment-free methods based on ML (Table 3). In fact, to our knowledge, BepiBlast, is the only tool that implements an alignment-based module designed and validated for the specific task of predicting linear B cell epitopes. However, it is worth noting that alignment-based approaches, similar to those implemented by BepiBlast, have been used to identify similarity between antigens and to detect antigen cross-reactivity and/or molecular mimicry^38,39^. Relevant examples of tools that have been released to detect molecular mimicry using alignment-based approaches are EPITOPEDIA^40^ and CE-BLAST^39^.

**Table 3:** Comparison of available web-based tools for predicting linear B cell epitopes.

Tool	Algorithm	Training dataset		Validation	URL	Reference
		B cell epitopes	Non-B cell epitopes			
BepiBlast	BLAST	62,730	–	X, I	http://imath.med.ucm.es/bepiblast/	–
Bceps	Support vector machine	555	555 (a)	X, I, E	http://imath.med.ucm.es/bceps/	^18^
BepiPred 2.0^a^	Random forest	3542	36,785	X, I, E	https://services.healthtech.dtu.dk/service.php?BepiPred-2.0	^20^
LBtope^b^	Support vector machine	14,876	23,321 (b)	X, I	https://webs.iiitd.edu.in/raghava/lbtope/	^17^
IBCE-EL	Random tree with boosting	4440	5485 (b)	X, I	http://www.thegleelab.org/iBCE-EL/	^28^
DLBEpitope	Deep neural network	22,012	201,563 (b)	X, I	http://ccb1.bmi.ac.cn:81/dlbepitope/index.php?	^15^
ILBE	Random Forest	4440	5485 (b)	X, I	http://kurata14.bio.kyutech.ac.jp/iLBE/	^41^
ABCPred	Neural network	700	700 (a)	X, I	https://webs.iiitd.edu.in/raghava/abcpred/	^14^
BCPREDS	Support vector machine	701	701 (a)	X, I, E	http://ailab.ist.psu.edu/bcpred/	^32^
SVMtrip	Support vector machine	4925	4925 (b)	X	http://sysbio.unl.edu/SVMTriP/prediction.php	^16^

For each tool, table reports the underlying algorithm; the number of B and non-B cell epitopes for model building; the method used for validation (X: cross-validation; I: independent dataset; E: case example); the URL of the tool and the reference. The letter between parenthesis indicates the type of non-B cell epitopes in the training dataset: a, random peptide sequences; b, peptide sequences with reported negative B cell epitope assays. ^a^For BepiPred, B and non-B cell epitope figures correspond to antigen residues that in the tertiary structure of antibody-antigen complexes contact the antibody or not, respectively. ^b^Data for default model in LBtope.

Compared with other tools, BepiBlast stands out for relying on the largest collection of known B cell epitopes without non-B cell epitopes. The absence of non-B cell epitopes may limit the chance for over fitting that particularly affect to ML-based methods^42^. Unlike competing tools, BepiBlast can also report if predicted B cell epitopes come from BLAST hits to neutralizing B cell epitopes as well as the accessibility and flexibility of B cell epitopes. Antibodies generated against predicted linear B cell epitopes do often fail to recognize the native protein, but this can be compensated by selecting B cell epitopes with enhanced flexibility and solvent accessibility^18,38,43,44^.

## Conclusions

We have shown that sequence similarity to available B cell epitope sequences poses a valid and advantageous approach to predict B cell epitopes on nominal antigens. We have enabled such predictions for free public use at BepiBlast.

## Methods

### B cell epitopes

Linear B cell epitopes were extracted from IEDB^25,26^. Only experimentally verified B cell epitopes with positive assays were considered and all sources were considered. An independent set of known linear B cell epitopes was downloaded from the BCIPEP database^27^. Only B cell epitopes with a size between 8 and 25 residues were considered and CD-HIT^45^ was used to reduce sequence redundancy, discarding sequences with identity ≥ 90%. B cell epitopes obtained from IEDB and BCIPEP were distinct.

### Non-B cell epitopes

Two types of non-B cell epitopes were considered in this study. A type of non-B cell epitopes consisted of random peptide sequences generated using the amino acid composition of proteins in the SWISSPROT database^29,30^. Length distribution of these non-B cell epitopes was fixed to resemble that of known B cell epitopes obtained from IEDB. The other type of non-B cell epitopes consisted of peptides with negative antibody recognition assays and size between 8 and 25 residues that were obtained from IEDB. All non-B cell epitopes were subjected to sequence redundancy using CD-HIT so that amino acid sequence identity was < 90%.

### Sequence similarity analysis

Sequence similarity was analyzed after pairwise sequence alignments generated using the Needleman–Wunsch global alignment algorithm implemented by the needle application of the *Biopython* package^46^. As we described elsewhere^47^, to obtain a measure of average sequence similarity in a dataset, all sequences were aligned pairwise but with themselves (for a dataset with N sequences there will be N × N − 1 alignments), identities were obtained for each alignment and the average identity was computed.

### Evaluation of BLAST-based predictions of B cell epitopes

B epitope predictions were assessed after BLAST^24^ searches to a BLAST formatted database consisting of B cell epitopes obtained from IEDB. Under this approach, any peptide sequence query with at least an ungapped hit with length ≥ 8 and identity ≥ 80% to the database was considered as a B cell epitope. BLAST-based predictions of B cell epitopes were evaluated under tenfold cross-validation, considering datasets of B cell and non-B cell epitopes with equal number of sequences. For each round of cross-validation, BLAST formatted databases were generated upon 90% of all B cell epitope sequences and used as a target database for BLAST testing of 10% of the remaining B cell epitopes as well as 10% of non-B cell epitopes. B cell and non-B cell epitopes with at least one hit (ungapped, length ≥ 8 and identity ≥ 80%) to the target database were considered as true and false positives (TP and FP), respectively, while non-B cell and B cell epitopes with no hits were considered as true and false negatives (TN and FN), respectively. Sensitivity (SE), specificity (SP), accuracy (ACC) and the Matthews correlation coefficient (MCC) were computed using Eqs. (1), (2), (3) and (4), respectively.

1 $$SE=\frac{TP}{TP+FN} \times 100,$$

2 $$SP=\frac{TN}{TN+FP} \times 100,$$

3 $$ACC=\frac{(TP+TN)}{(TP+FP+TN+FN)} \times 100,$$

4 $$MCC=\frac{\left(TP \times TN\right)-(FP \times FN)}{\sqrt{(TN+FN)(TP+FN)(TN+FP)(TP+FP)}}.$$

These same criteria and parameters were used to evaluate BLAST-based B cell epitope predictions on independent datasets.

### Prediction of linear B cell epitopes with freely available tools

For comparative analysis, linear B cell epitopes were predicted using the web-based tools IBCE-EL^28^ and LBtope^17^, available at http://www.thegleelab.org/iBCE-EL/ and https://webs.iiitd.edu.in/raghava/lbtope/peptide.php, respectively. LBtope predictions were carried out selecting the default model labeled as “LBtope_Variable (original dataset)” and peptides with probability higher than 0.6 were considered B cell epitopes, as suggested by the tool. BCE-EL predictions were also carried out with default settings, considering peptides labeled by the tool as “BCE” as B cell epitopes. B cell epitopes were also predicted using a standalone version of BepiPred (Bepipred 2.0)^19,20^. BepiPred assigns B cell epitope propensities per residue and average B cell epitope scores were computed. Peptide sequences with scores ≥ 0.5 were considered as B cell epitopes.

### BLAST-based detection of B cell epitopes in protein sequences

B cell epitopes in protein query sequences were detected after BLAST searches to a database consisting of known linear B cell epitopes using the standalone version of BLAST with default settings. Protein query fragments from hits with length ≥ 8, identity ≥ 80% and no gaps were considered as B cell epitopes. Since BLAST searches often produce various overlapping hits, we devised a system to simplify the results and identify B cell epitope cores. To that end, the occurrence of protein residues in hits is first tallied up (*B*). Residues that are not included in any hit are assigned *B* = 0. Subsequently, normalized B values, *Z*_*B*_, are computed using Eq. (5).

5 $${Z}_{B}=\frac{B- {\mu }_{B}}{{\partial }_{B}}.$$

*B* is the tallied up occurrence of a particular residue in B cell epitope hits, *µ*_*B*_ is the mean of B of all protein residues and *∂*_*B*_ is its standard deviation. *Z*_*B*_ values were then used to identify B cell epitope cores as those consisting of 8 residues or more (those matching overlapping B cell epitope hits) with *Z*_*B*_ values ≥ than flanking residues.

### Other procedures

B cell epitope scores were computed as bit scores upon BLAST hit alignments using the BLOSUM62 substitution matrix^48^. Relative solvent accessibility (RSA) and normalized B values—used as a measure of flexibility—per residue were predicted for the entire protein query sequences using NetSurfP^49^ and profBval^50^, respectively, and measures of epitope accessibility and epitope flexibility consisted of average values computed from the corresponding epitope residue values.

### Web implementation

BLAST-based B cell epitope predictions were implemented for free public use on the Web using a Python CGI (Common Gateway Interface) script that executes BLAST searches on user-provided input data and returns the predicted B cell epitopes to the browser, along with epitope annotation information from IEDB. The front-end web interface was developed using Hyper Text Markup Language (HTML) in combination with Cascading Style Sheets (CSS) and JavaScript. Web page administration is done using Apache HTTP Server (https://httpd.apache.org).

## Supplementary Information

Supplementary Information.

## Data Availability

All data generated or analysed during this study are included in this published article and its Supplementary information files.
